# Multi-phase cycle coding for SSVEP based brain-computer interfaces

**DOI:** 10.1186/1475-925X-14-5

**Published:** 2015-01-16

**Authors:** Jijun Tong, Danhua Zhu

**Affiliations:** School of Information Science and Technology, Zhejiang Sci-Tech University, Hangzhou, 310018 China; State Key Laboratory of Diagnosis and Treatment of Infectious Diseases, Collaborative Innovation Center for Diagnosis and Treatment of Infectious Diseases, The First Affiliated Hospital Of Zhejiang University, Hangzhou, 310003 China

**Keywords:** Brain computers interfaces, Cyclic coding, SSVEP, Phase synchrony

## Abstract

**Background:**

Brain-computer interfaces (BCIs) based on Steady State Visual Evoked Potential (SSVEP) have attracted more and more attentions for their short time response and high information transfer rate (ITR). The use of a high stimulation frequency (from 30 Hz to 40 Hz) is more comfortable for users and can avoid the amplitude-frequency problem, but the number of available phases for stimulation source is limited. To circumvent this deficiency, a novel protocol named Multi-Phase Cycle Coding (MPCC) for SSVEP-based BCIs was proposed in the present study.

**Methods:**

In MPCC, each target is coded by a block word that includes a series of cyclic codewords, and each block word is corresponding to a certain flickering visual stimulus, which is a combination of multiple phases from an available phase set and flickers at single frequency. The methods of generating block code and extracting phase were presented and experiments were performed to investigate the feasibility of MPCC.

**Results:**

The optimal stimulation frequency was subject-specific, and the optimal duration was longer than 0.5 s. The BCI system with MPCC could achieve average discrimination accuracy 93.51 ± 5.62% and information transfer rate 33.77 ± 8.67%.

**Conclusions:**

The MPCC has the error correction ability, can effectively increase the encoded targets and improve the performance of the system. Therefore, the MPCC is promising for practical BCIs.

## Background

Brain-computer interface (BCI) is the collaboration between brain and devices that enables signals from brain to direct some external activities. The interface builds a direct communication pathway between brain and the objects to be controlled. This article presents a BCI system using Steady State Visual Evoked Potential (SSVEP) as neural source. SSVEP refers to the response of cerebral cortex to a repetitive visual stimulus (RVS) oscillating at a constant frequency, and can be characterized by peaks at the fundamental frequency and its harmonics in the power spectral density (PSD) of recorded EEG signals [1]. It is an effective neural source compared to those in other BCI systems (e.g. motor imagery [2, 3], P300 [4, 5], and slow cortical potentials [6]), since it can achieve higher information transfer rate (ITR) with shorter time response [7, 8].

Till now, three methods to implement SSVEP-based BCIs have been proposed. The first method, and also the most popular one, is to encode the targets with frequencies, where the targets are encoded with single frequency [9–12] or the combination of dual or multi frequencies [13–16]. The second one is to encode the targets with phases, where each target oscillates at one fixed frequency but different phases [17–20]. The third one is to encode the targets with both phases and frequencies [21]. The first and third methods need at least two frequencies, which will induce amplitude-frequency problem [22], i.e. the SSVEP signal will decrease due to the competition in the corresponding cerebral cortex when the subject focuses on several stimuli flickering at two or more frequencies [23]. Instead the second method only needs one frequency, which can effectively avoid the selection and identification of multiple frequencies. However, the number of available phases is limited, e.g., no more than eight phases can be used at a single frequency [18, 21]. Another important issue to be considered is that which frequency should be selected. The repetitive visual stimulus at high frequency (larger than 30 Hz) is safer and more comfortable for users and causes less visual fatigue. However,only few frequencies can elicit sufficiently strong SSVEPs for BCI purposes in the high frequency range (30 Hz ~ 40 Hz) [24].

To overcome these issues, this article proposed a novel protocol termed as Multiple Phases Cycle Coding (MPCC) for SSVEP-based BCIs. In MPCC, the stimulation source flickers at high frequency and each target is coded by a block code that includes a series of cyclic codes. Each codeword is corresponding to a certain flickering visual stimulus mode, which is a combination of several phases from an available phase set. This method can encode more targets and improve the system performance with the ability of error correction. Besides, it also increases the user’s comfort and safety.

The paper is organized as follows. Method provides a detailed description about MPCC, the stimulator, the experiment protocol, and also the signal processing methods. Results presents the results. Discussion is a general discussion of the results.

## Method

### Multiple Phases Cycle Coding (MPCC)

#### The basic principle of MPCC

In SSVEP-based BCIs, the stimulus source flickers at a certain pattern and users pay their attentions on the stimulus to produce SSVEP signal, which will further be used to generate the commands. It is generally believed the latency of individual’s SSVEP is relatively stable, which means the SSVEP is phased-locked to the visual stimulus [25–27]. In addition, the moment of focusing on a stimulus is dependent upon user, thus the set of all cyclic shifts of a sequence was used as a block word other than associating a sequence with a single stimulus.

A cyclic code was divided into several block codes, where the circular shifts of each codeword gave another word that belongs to the code. Thus, the block code is also named as cyclic class.

In this article, to achieve the MPCC, the cyclic code introduced in [28] was used, where the targets were coded by the predefined phases cycle by cycle, and each cycle was divided into several coding epochs that were corresponding to a flickering visual stimulus with certain phase.

**Figure 1:**
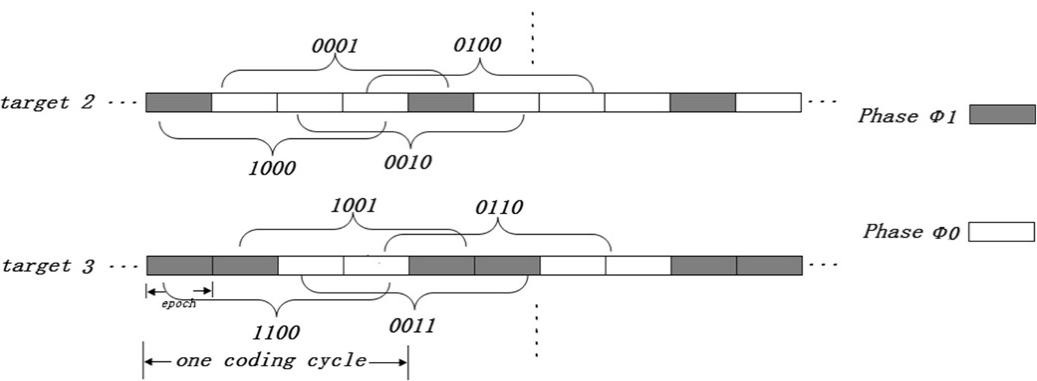
**The paradigm of Multiple Phases Cycle Coding (MPCC), where the gray bar denotes the coding stimulus phase Φ1, while the white bar denotes the coding stimulus phase Φ0.**

It is noted that two phases can code six targets with the code length of 4 after cycle coding, compared to 16 (=2^4^) targets with the same conditions in the traditional coding method. With the cycle coding method, though the available targets decrease, a user only needs focusing his attention on the stimulus without concerning the time synchronous and time breaks between cycles, and any decoded code words in one sequence can be the target, as shown in Figure 1. Another advantage is that they are error-correction codes, which have algebraic properties that are convenient for efficient error detection and correction, and consequently enhance the discrimination accuracy.

#### The generation of cyclic class and the detection of code word

Several ways to generate the cycle error-correction code have been proposed, in the present study, considering that the code length is usually short in the SSVEP-based BCI, the Gilbert exhaustive search algorithm, which is based on the Hamming distance, is used [29, 30].

##### Class leader

The class leader represents a cyclic class. It also can decrease the word candidates and consequently increase the speed of searching for a good code.

In a cyclic shift set *m*, the class leader is defined as the unique sequence that is the smallest one in a lexicographical ordering. For example, given


In lexicographical order, we can achieve 01011 < 01101 < 10101 < 10110 < 11010, so the class leader is 01011. An algorithm to create cyclic classes through finding class leaders is described as follows. Considering all sequence *x*^*N*^ in lexicographical order,

i. Starting with the smallest sequence *x*^*N*^ = 0^*N*^;
ii. *x*^*N*^ must be a class leader, since all lexicographically smaller sequences have already been considered;
iii. Removing all cyclic shifts of *x*^*N*^ from the ordered list;
iv. If the list is empty then stop, else let *x*^*N*^ be the smallest sequence in the ordered list and continue with the step ii.

The example of class leaders obtained by the algorithm with A as {0, 1, 2} and the word length as 5 is listed as follows. There are 51 cyclic classes and each of them can be associated to a stimulus source. But in order to obtain a good performance of classifying each other, a good error-correcting code is needed.

{00000_3_, 00001_3_, 00002_3_, 00011_3_, 00012_3_, 00021_3_, 00022_3_, 00101_3_, 00102_3_, 00111_3_, 00112_3_, 00121_3_, 00122_3_, 00201_3_, 00202_3_, 00211_3_, 00212_3_, 00221_3_, 00222_3_, 01011_3_, 01012_3_, 01021_3_, 01022_3_, 01102_3_, 01111_3_, 01112_3_, 01121_3_, 01122_3_, 01202_3_, 01211_3_, 01212_3_, 01221_3_, 01222_3_, 02021_3_, 02022_3_, 02111_3_, 02112_3_, 02121_3_, 02122_3_, 02211_3_, 02212_3_, 02221_3_, 02222_3_, 11111_3_, 11112_3_, 11122_3_, 11212_3_, 11222_3_, 12122_3_, 12222_3_, 22222_3_}.

##### The construction of an error-correction code

In general, constructing a good error-correction code is challenging. In case of SSVEP-based BCIs, however, the code length is short and thus an exhaustive search method for good codes can be used. The search method is based on the Cyclic Hamming Distance (CHD) between classes. In information theory, the Hamming distance between two strings of equal length is the number of positions at which the corresponding symbols are different. Here the Cyclic Hamming Distance (CHD) is defined as the minimum Hamming distance between the cyclic shifted versions of two class leaders.

Given a minimum Cyclic Hamming Distance (CHD), a word length N and a symbol set A, a list of all cyclic classes can be found with Gilbert construction from the class leaders. L is the class list in which the distance between each other is equal to or bigger than d. W is a class list in which the classes have a minimum cyclic distance of d. After that, the search of the cyclic code can be performed using the recursive algorithm as shown below, where the function ‘cyclicdistance’ calculates the Cyclic Hamming Distance (CHD) between two class leaders.


##### Detection of word

The Minimum Distance Detection (MDD) is used to detect the code word. It compares the Hamming distance between the detected code word and all block codes, and the code word that has the minimum distance is considered as the target code word.

Cyclic codes have the ability to correct errors. From the concept of Hamming distance a cyclic code with a minimum Hamming distance 2 t + 1 can correct any t errors.

### Implementation of MPCC in SSVEP-based BCIs

As described above when MPCC is used to encode targets, each symbol is corresponding to a feature such as a frequency or a phase of SSVEP. Considering the SSVEP characteristics of frequency-dependence, we apply several phases at one frequency. Then each target of our system is encoded with the combination of these phases.

#### SSVEP feature extraction

**Figure 2:**
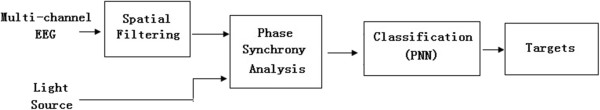
**The procedure of target recognition.**

To obtain the SSVEP phases effectively, a signal processing method based on multi-channel EEG fusion is proposed, where pattern recognition methods are used to identify the subject's intension. Figure 2 illustrates this procedure. In this section, the methods of spatial filtering and phase synchrony analysis based on the Hilbert transform are described [24, 31].

##### The SSVEP enhancement using spatial filtering

An EEG epoch X can be written as a *T* × *N* matrix with the columns as the signals of N recorded electrodes and the rows as the T time samples in the epoch *x*_*i*_, *i* = 1, …, *N*. The spatially filtered signal x_*w*_ can be written as a linear combination of the {*x*_*i*_}.


where
1

The spatial filter coefficients are estimated so that the ratio between the SSVEP and background activity is maximized [32]. The maximal contrast combination method in [32] is used to estimate w at an EEG epoch basis as follows:
2

Where *Q* = *S*(*S* ' *S*)^− 1^*S* ', *S*' is a matrix with the columns as the signals in the set
3

These are sinusoidal signals at the stimulation frequency f and its harmonics H. In this study, only the stimulation frequency, i.e., H = 1, is considered, since the high stimulation frequencies (>30 Hz) is used and the EEG spectral content is restricted to 60 Hz.

In equation (2), the per-epoch covariance matrices X'X and (X ‒ QX) ' (X ‒ QX) are used to estimate the SSVEP activities and the background activities, respectively. A better and more stable estimation of the covariance matrix can be obtained if the covariance matrices of several EEG epochs are averaged. Thus we propose to estimate the optimum spatial filter as follows:
4

Where X_*k*_ is the k-th EEG epoch and *K* is the total number of epochs that are considered.

##### Phase synchrony analysis

The phase is estimated through phase synchrony analysis. It first calculates the instantaneous phase difference *δ*_*f*_ (t) between z_*f*_ (t) and the stimulation signal filtered with the peak filter centered at *f*. This signal is denoted as *l*_*f*_ (t).

The analytical signals associated with _*f*_ (t) and *l*_*f*_ (t), *A*{*z*_*f*_}(*t*) and *A*{*l*_*f*_}(*t*) respectively can be written as:
5

where H{*z*_*f*_}(t) and H{*l*_*f*_}(t) are the Hilbert transform of z_*f*_ (t) and *l*_*f*_ (t), *R*{•} and Θ{•} are the instantaneous amplitude and phase, respectively. Thus, the instantaneous phase difference *δ*_*f*_ (t) is equal to *Θ*{*z*_*f*_}(t) − *Θ*{*l*_*f*_}(t). The phase is estimated as the median of *δ*_*f*_ (t) in a given time window, e.g. the epoch duration.

##### Feature vector classification

A probabilistic neural network (PNN) is used to identify the user's focus of attention from the feature vector. A PNN is a radial basis network that estimates the probability density function of each class from labeled training data [33] using the Parzen window technique.

Considering the individual differences involved and the trained classifier for each subject, 27 segments of EEG were used as training and testing dataset, and leave-one-out cross validation was used.

#### Selection of optimal stimulus frequency

The stimulation frequency eliciting the strongest SSVEP response (optimal stimulation frequency) is user dependent [34], and higher frequencies cause less visual fatigue. Therefore, we implemented a procedure aiming at selecting the optimal stimulation frequency in the range from 32 to 40 Hz [35].

#### Optimization of phase duration

Considering that the duration of one command cannot be too long, it is necessary to select an optimal duration of a phase. In MPCC, the stimulation frequency is kept unchanged, while the phase is determined by the symbol in the code word. The phase is expressed as follows:
6

where *ϕ*_*j*_ is an arbitrary one of a phase set, *t*_*j*_ is the onset of the stimulus, ∆*t* is the duration of the stimulus with this phase. The duration ∆*t* is very important, since its too short will make SSVEP phase transition occur before achieving stability, and its too long will make the single code word lasting too long and the ITR decrease.

#### Performance evaluation

To evaluate the performance of BCI, consistent criteria are necessary. The most popular criterion is the information transfer rate (ITR), which measures the information transmitted by the system in unit time and is calculated based on the popular bit rate definition [36]. Specifically, the bit rate R and ITR for N classes and classification accuracy *p* are defined as followes:
78

Where τ is the average time (in seconds) needed to identify one symbol.

### Experimental protocol

In this study, two green LED (diameter = 5 cm) flickering simultaneously driven by square wave currents, which were rendered from two Agilent Function Generators (Model: 33220A), were used as the stimulation sources. The distance between two LED boxes was around 30 cm.

These two LEDs flickered at the same frequency but with different phases. One LED had the unchanged phase *0* while the other had the changed phase (0, 2π/3, 4π/3). A photodiode was used to record the light source with the unchanged phase, which was used to extract the phase difference between the light signal and SSVEP using phase synchrony analysis.

Three phases (0, 2*π*/3, 4*π*/3) were used in the experiment, representing three symbols {0, 1, 2}. And there were 27 (= 3^3^) combinations; each one corresponded to one stimulus cycle. Each subject received 27 stimuli with the different phase duration ∆*t*. During the two adjacent visual stimuli, the subjects had 5–6 seconds to rest. The subjects were required to avoid movements or blinking during stimulus periods, while advised to blink during the resting period between two consecutive stimuli.

Eight subjects were recruited in this study. They were requested to seat in front of a lamp positioned approximately 0.5 meter away from their eyes. All subjects had no neurological disease, especially epilepsy. They had signed an informed consent before engaging in this study and had the right to quit at any time. The scope of ∆*t* was randomly selected from 0.2 to 1.0 with the step of 0.1.

EEG signals were recorded using a BioSemi Active-two EEG acquisition device in a normal office environment with curtain closed and lights on. Thirty-two electrodes were placed according to the international 10–20 system. The impedance between scalp and electrodes was kept below 5 kΩ. The sampling frequency was 2048 Hz.

## Results

During offline analysis, all experimental data were preprocessed firstly. They were down-sampled to 256 Hz, Cz was used as the re-referenced electrode and the electrodes O1, O2, Oz, PO3, PO4, P3, P4 and Pz were used for spatial filtering, since the occipital and parietal regions had the strongest response of SSVEP [1]. In the spatial filtering and phase extraction, the time window was the duration of one phase and there was no overlap between two windows.

### Determination of stimulation duration

**Figure 3:**
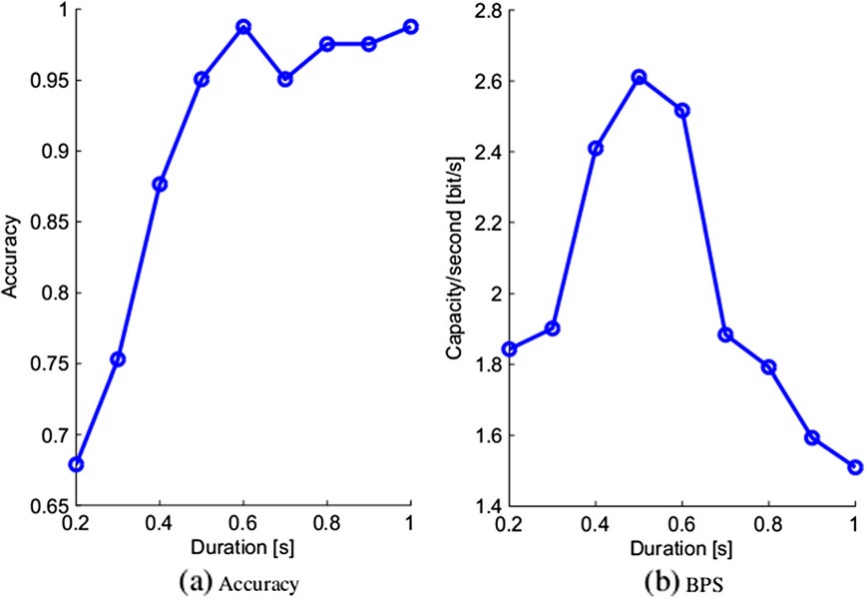
**The relationship between classification accuracy, maximum amount of information transfer per unit time and stimulation duration. (a)** The classification accuracy as a function of stimulation duration. **(b)** The bit rate as a function of stimulation duration.

Figure 3 shows the discrimination accuracy and bit rate per second for different stimulation durations from subject S1. As shown in Figure 3(a), the accuracy increases at first and then becomes stable. So the optimal durations greater than 0.5 s are all available.

In contrast, Figure 3(b) shows the BPS of different durations. The BPS increases at first and reaches a maximum of 2.61 bits/second, and then it decreases. The duration corresponding to the maxima is the optimal one. The third column of Table 1 shows the optimal durations of eight subjects, it also shows that the optimal duration depends on subjects and is longer than 0.5 second. The classification accuracies and BPSs are shown in the fourth and fifth columns, respectively. It also demonstrates that the spatial filter and phase synchrony analysis methods presented are effective.

**Table 1:** **The optimal frequency, duration, accuracy and BPS of all subjects**

Subjects	Optimal frequency (Hz)	Optimal duration(s)	Discrimination accuracy	BPS(bit/symbol)
S1	40	0.5	95.06%	2.61
S2	40	0.7	95.06%	1.88
S3	40	1.0	90.12%	1.06
S4	39	0.6	82.72%	1.40
S5	40	0.7	95.06%	1.83
S6	37	0.7	88.89%	1.47
S7	37	0.6	90.12%	1.81
S8	37	0.6	95.06%	2.13
Average(Mean ± SD)			91.51 ± 4.45%	1.77 ± 0.48

### The performance test of cyclic word

During the experiment, each phase was repeated 27 times for each subject, and each symbol of code word was chosen randomly from the data set. Then we combined them according to the code word in Table 2 and repeated it 20 times.

**Table 2:** **Six code examples of different word length N, minimal word distance d**_**min**_ **and a symbol set {0,1,2}**

	N	d _min_	Number of code words	Code word
C1	3	2	5	{000, 012, 021, 111, 222}
C2	4	2	9	{0000, 0011, 0022, 0101, 0202, 1111, 1122, 1212, 2222}
C3	5	3	6	{0000, 0011, 0022, 0101, 0202, 1111, 1122, 1212, 2222}
C4	6	3	10	
C5	8	4	16	
C6	10	5	14	

Table 3 shows the discrimination accuracy of eight subjects for all encoded word. It is observed that C6 has the highest accuracy except S7, since it has the greatest code distance and code length compared with other code words, which mean it could correct more errors. Theoretically, if the code length is long enough, it is possible to obtain 100% accuracy for detecting a code word. But in the practical BCI system, it is inappropriate to adopt too long code because it needs more time to identify the target. The code C6 is not suitable for S3, because the phase duration is 1 s and the system needs 10s to identify one command, but it is still suitable for S1 because the phase duration is 0.5 s and the system needs 5 s to identify one command.

**Table 3:** **The discrimination accuracies of the 8 subjects with different code word**

Subject	C1	C2	C3	C4	C5	C6
S1	91.99%	91.99%	97.92%	97.35%	96.31%	99.25%
S2	87.52%	85.80%	97.26%	96.60%	96.15%	98.71%
S3	77.44%	72.58%	91.94%	89.45%	85.79%	94.59%
S4	69.05%	63.09%	81.95%	76.85%	71.48%	84.77%
S5	91.83%	88.67%	97.96%	97.67%	97.35%	99.45%
S6	79.24%	72.22%	90.28%	88.35%	86.80%	93.25%
S7	78.99%	74.48%	92.69%	56.08%	46.87%	61.50%
S8	92.45%	92.12%	98.04%	97.67%	97.70%	99.54%
Average (Mean ± SD)	83.56 ± 8.63%	80.12 ± 10.90%	93.51 ± 5.62%	87.51 ± 14.60%	84.81 ± 17.75%	91.38 ± 13.08%

Table 4 shows the IRTs of eight subjects for all encoded words. The performance of S3 and S7 is worse than others. For S3, it may because the duration of a single phase is one second, longer than the other subjects, while as for S7, it should be the low code word discrimination accuracy.

**Table 4:** **The ITR (bits/minute) of the 8 subjects with different code word**

Subject	C1	C2	C3	C4	C5	C6
S1	47.81	36.96	49.55	38.63	29.02	32.51
S2	34.18	24.40	35.13	27.53	20.84	23.22
S3	18.87	13.60	21.20	16.84	12.51	15.33
S4	20.20	14.74	26.25	20.60	14.87	20.79
S5	30.79	24.50	34.82	27.19	20.25	23.09
S6	23.21	17.42	29.20	22.89	17.09	21.12
S7	23.21	16.62	33.96	9.41	5.79	10.15
S8	35.70	28.17	40.01	30.88	23.45	26.77
Average (Mean ± SD)	29.25 ± 9.82	22.05 ± 7.99	33.77 ± 8.67	24.25 ± 8.95	17.98 ± 7.11	21.62 ± 6.78

The results show that the discrimination performance is different among different subjects and different coding codewords, indicating that each subject has its own optimal cycle coding.

## Discussion

This study presents a SSVEP-based BCI using Multi-Phase Cycle Coding. The proposed method encodes each target with a block word, which comprises of a series of cyclic codewords, and each block word is corresponding to a certain visual stimulus source that flickers at a single frequency and is combined by multiple phases from an available phase set. Compared to other SSVEP-based BCI systems, the present BCI system has significant advantages.

First of all, the system only utilizes single flickering frequency and avoids the amplitude-frequency problem. It uses the high stimulation frequency (30 Hz < f < 40 Hz), which is safer and more comfortable for users, causes less visual fatigue, and avoids the interference of low frequency environmental noise and some brain rhythms. Furthermore, considering the optimal frequency is subject dependent, it selects the optimal stimulation frequency for each subject.

Secondly, the proposed protocol can encode multiple targets. In MPCC, each target is coded by a block word that comprises of a series of cyclic codewords. The number of code words depends on the number of symbols (the phases set of SSVEP), the code length and minimum Hamming distance. It is obvious that the available code words have positive relationship with symbols and code length, and negative relationship with code distance. Theoretically, the proposed method can encode countless targets through increasing the code length and symbol set. However, the symbol number cannot be arbitrary large since eight phases can be used at most under one frequency. Also the code length cannot unlimited grow since the time for users to identify one command is limited. For example, when the code length reaches 6, the code word duration of subject S3 is 6 s, it is a bit longer for user’s attention and BCI system to produce one command.

The other effective way to get more output commands is encoding a series of single commands to represent a final target. But it requires the user to remember the command sequence mode and intervals between two commands, so it will decreases the ITR.

Thirdly, the proposed protocol has the error-correction ability and thus enhances the system’s discrimination accuracy. Compared with the other studies, it has similar or better performance in discrimination accuracy. The average discrimination accuracy is 91.38 ± 13.08% with code C6 while 83.56 ± 8.63% with code C1. In contrast, Wong et al. achieved the discrimination accuracy of 90.69% (from channel Oz) [37], Jia et al. reached 85% (from channels Oz-POz) [21] and Lee et al. reached 83.49 ± 10.4% (from channel Oz) [18].

The error-correction ability depends on the minimal word distance **d**_**min**_, the bigger d_min_ has better error-correction ability. As shown in Table 3, the word C6, which has the maximal d_min_, has the best performance except for subject S7. But with the increase of d_min_, the available number of code words will decrease and the code length will increase. However, it is harmful to coding and unacceptable for a practical BCI system. Thus it is a tradeoff among the code length, the command numbers and ability of error correction.

Furthermore, the system reliability should be concerned in a practical BCI system. The wrong command will bring greater danger than the miss detection of a command (i.e., allow the system to standby). For example, when a user controls an electric wheelchair, a wrong command will cause an error in the traveling direction, which could cause great harm to the user. There are two available ways to improve the reliability of system.

The first one is strictly limiting the distance between the sequence of symbols and code words to zero in detecting the code word, and the system will has less possibility to output error commands. In this article, the minimum distance detection (MDD), which was based on the cyclic Hamming distance and the detected word was the one that has the smallest distance with any words in a code group, was used to detect the code word, the distance between the object code word and sequence may be bigger than zero. Therefore, it is necessary to limit the distance to zero before it is transferred into practical applications in the future studies.

Another method to improve the system reliability is to use the additional features before generating the command. In recent years some researchers have combined two or more brain activity patterns or input signal sources to create a more reliable BCI system, which is the so called hybrid BCI system [38, 39]. Notably, the error-related potentials (ErrPs) have been used as second level information to correct system decisions, which are certain types of ERPs observed in the EEG signals when the user is aware of erroneous behavior [40]. ErrPs can be reliably decoded on a single trial basis, thus enabling their application in BCI systems as a means to improve the system’s performance.

The present study mainly focuses on the design and implementation of cyclic code. However, there are many ways of cyclic code such as Fire code, Golay code, BCH (Bose Chaudhuri-Hocguenghem) code, and so on. The future work will search for optimal and proper coding method and apply them in the BCI systems.

## Conclusions

In this paper, a new BCI protocol named MPCC is proposed to overcome the restriction of available phase number under one frequency and encodes more targets. It also has the ability of error correction and can enhance the decoding accuracy. The experiment results indicate that each subject has its own optimal stimulation frequency and duration, and MPCC has satisfactory discrimination accuracy and ITR compared with the previous studies, suggesting it is a promising choice in the future BCI systems.

## References

[1] Regan D, Human Brain Electrophysiology (1990). Evoked potential and evoked magnetic fields in science and medicine. J Clin Neurophysiol.

[2] Kaiser V, Bauernfeind G, Kreilinger A, Kaufmann T, Kubler A, Neuper C (2014). Cortical effects of user training in a motor imagery based brain–computer interface measured by fNIRS and EEG. Neuroimage.

[3] Liu Y, Li MF, Zhang H, Wang H, Li JH, Jia J (2014). A tensor-based scheme for stroke patients’ motor imagery EEG analysis in BCI-FES rehabilitation training. J Neurosci Methods.

[4] Farwell LA, Donchin E (1988). Talking off the top of your head: toward a mental prosthesis utilizing event-related brain potentials. Electroencephalogr Clin Neurophysiol.

[5] Kapeller C, Ortner R, Krausz G, Bruckner M, Allison BZ, Guger C (2014). Toward multi-brain communication: collaborative spelling with a P300 BCI. Foundations of augmented. Cognition.

[6] Birbaumer N, Kubler A, Ghanayim N, Hinterberger T, Perelmouter J, Kaiser J (2000). The thought translation device for completely paralyzed patients. IEEE Trans Rehabil Eng.

[7] Cheng M, Gao X, Gao S (2002). Design and implementation of a brain-computer interface with high transfer rates. IEEE Transact Biomed Eng.

[8] Wu ZH (2014). Using canonical correlation method to extract SSVEP at one channel. Proc Second Int Conf Commun Signal Proc Syst.

[9] Gao X, Xu D, Cheng M, Gao S (2003). A BCI-based environmental controller for the motion-disabled. IEEE Trans Neural Syst Rehabil Eng.

[10] Friman O, Luth T, Volosyak I, Graser A (2007). Spelling with steady-state visual evoked potentials. 3rd International IEEE/EMBS Conference on Neural Engineering.

[11] Lalor EC, Kelly SP, Finucane C, Burke R, Smith R, Reilly RB (2005). Steady-state VEP-based brain-computer interface control in an immersive 3D gaming environment. EURASIP J Appl Signal Process.

[12] Sun F, Hu D, Liu H, Chen G, Song D, Liao L, Sun F, Hu D, Liu H (2014). A multi-channel SSVEP-based brain–computer interface using a canonical correlation analysis in the frequency domain. Foundations and practical applications of cognitive systems and information processing.

[13] Srihari Mukesh TM, Jaganathan V, Ramasubba Reddy M (2006). A novel multiple frequency stimulation method for steady state VEP based brain computer interfaces. Physiol Meas.

[14] Shyu K, Lee P, Liu Y, Sie J (2010). Dual-frequency steady-state visual evoked potential for brain computer interface. Neurosci Lett.

[15] Zhang Y, Xu P, Liu T, Hu J, Zhang R, Yao D (2012). Multiple frequencies sequential coding for SSVEP-based brain-computer interface. Plos One.

[16] Hwang HJ, Hwan KD, Han CH, Im CH (2013). A new dual-frequency stimulation method to increase the number of visual stimuli for multi-class SSVEP-based brain-computer interface (BCI). Brain Res.

[17] Wang Y, Gao X, Hong B, Jia C, Gao S (2008). Brain-computer interfaces based on visual evoked potentials. IEEE Eng Med Biol Mag.

[18] Lee PL, Sie JJ, Liu YJ, Wu CH, Lee MH, Shu CH (2010). An SSVEP-actuated brain computer interface using phase-tagged flickering sequences: a cursor system. Ann Biomed Eng.

[19] Manyakov NV, Chumerin N, Van Hulle MM (2012). Multichannel decoding for phase-coded SSVEP brain-computer interface. Int J Neural Syst.

[20] Yang QL, Wu PD (2014). 3D image control system based on phase-coding BCI.

[21] Jia C, Gao X, Hong B, Gao S (2011). Frequency and phase mixed coding in SSVEP-based brain-computer interface. IEEE Transact Biomed Eng.

[22] Parini S, Maggi L, Turconi AC, Andreoni G (2009). A robust and self-paced BCI system based on a four class SSVEP paradigm: algorithms and protocols for a high-transfer-rate direct brain communication. Intell Neurosc.

[23] Keitel C, Andersen SK, Muller MM (2010). Competitive effects on steady-state visual evoked potentials with frequencies in- and outside the alpha band. Exp Brain Res.

[24] Zhu D, Garcia-Molina G, Mihajlovic V, Aarts RM (2010). Phase synchrony analysis in SSVEP-based BCIs. 2nd Int Conf Comp Eng Technol Chengdu, China.

[25] Pastor MA, Artieda J, Arbizu J, Valencia M, Masdeu JC (2003). Human cerebral activation during steady-state visual-evoked responses. J Neurosci.

[26] Vialatte FB, Maurice M, Dauwels J, Cichocki A (2010). Steady-state visually evoked potentials: focus on essential paradigms and future perspectives. Progess Neurobiol.

[27] Yeh CL, Lee PL, Chen WM, Chang CY, Wu YT, Lan GY (2013). Improvement of classification accuracy in a phase-tagged steady-state visual evoked potential-based brain computer interface using multiclass support vector machine. BioMed Eng OnLine.

[28] Tjalkens T, Zhu DH (2012). The cyclic shift channel. Proceedings of the 2012 information theory and applications workshop.

[29] Gelbert EN (1952). A comparison of signal alphabets. Bell Syst Tech J.

[30] Nguyen GD (2005). Error-detection codes: algorithms and fast implementation. IEEE Transact Comp.

[31] Molina GG, Ibaňez D, Mihajlović V, Chestakov D (2009). Detection of high frequency steady state visual based brain-computer interfaces. 17th European Signal Processing Conference.

[32] Friman O, Volosyak I, Gräser A (2007). Multiple channel detection of steady-state visual evoked potentials for brain-computer interfaces. IEEE Transact Biomed Eng.

[33] Parzen E (1962). On estimation of a probability density function and mode. Ann Math Stat.

[34] Molina GG, Mihajlovic V (2010). Spatial filters to detect steady state visual evoked potentials elicited by high frequency stimulation: BCI application. J Biomed Eng.

[35] Zhu DH, Molina GG, Mihajlovic V, Arts RM (2011). Online BCI Implementation of high frequency phase modulated visual stimuli. Univ Access Hum Comp Interact Users Dive Lect Notes Comp Sci.

[36] Wolpaw JR, Birbaumer N, McFarland DJ, Pfurtscheller G, Vaughan TM (2002). Brain-computer interfaces for communication and control. Clin Neurophysiol.

[37] Wong CM, Wang BY, Wan F, Mak PU, Mak PI, Vai MI (2010). An improved phase-tagged stimuli generation method in steady-state visual evoked potential based brain-computer interface. 3rd International Conference on Biomedical Engineering and Informatics (BMEI 2010).

[38] Amiri S, Fazel-Rezai R, Asadpour V (2013). A review of hybrid brain-computer interface systems. Adv Hum Comp Interact.

[39] Yin E, Zhou ZT, Jiang J, Chen FL, Liu YD, Hu DW (2014). A speedy hybrid BCI spelling approach combining P300 and SSVEP. IEEE Transact Biomed Eng.

[40] Chavarriaga R, Sobolewski A, Millan JR (2014). Errare machinale est: the use of error-related potentials in brain-machine interfaces. Front Neurosci.

